# The energy landscape of magnetic materials

**DOI:** 10.1038/s41524-024-01310-w

**Published:** 2024-07-16

**Authors:** Louis Ponet, Enrico Di Lucente, Nicola Marzari

**Affiliations:** 1 Theory and Simulation of Materials (THEOS) and National Centre for Computational Design and Discovery of Novel Materials (MARVEL), École Polytechnique Fédérale de Lausanne, Lausanne, 1015 Switzerland; 2 Laboratory for Materials Simulations (LMS), Paul Scherrer Insititute, Villigen, 5232 Switzerland

**Keywords:** Electronic properties and materials, Electronic structure

## Abstract

Magnetic materials can display many solutions to the electronic-structure problem, corresponding to different local or global minima of the energy functional. In Hartree-Fock or density-functional theory different single-determinant solutions lead to different magnetizations, ionic oxidation states, hybridizations, and inter-site magnetic couplings. The vast majority of these states can be fingerprinted through their projection on the atomic orbitals of the magnetic ions. We have devised an approach that provides an effective control over these occupation matrices, allowing us to systematically explore the landscape of the potential energy surface. We showcase the emergence of a complex zoology of self-consistent states; even more so when semi-local density-functional theory is augmented - and typically made more accurate - by Hubbard corrections. Such extensive explorations allow to robustly identify the ground state of magnetic systems, and to assess the accuracy (or not) of current functionals and approximations.

## Introduction

Many scientific and technological advances in recent decades have been heralded by the discovery of magnetic phenomena^1,2^, and the identification of materials that exhibit them in an exploitable manner. In recent years, the field of spintronics, where electrons’ intrinsic spin represents an extra degree of freedom for electronic transport in addition to their charge state^3^, has come to the forefront as one of the next frontiers for technological progress. Investigations aim at enlarging the storage capacity of devices^4,5^, tuning the spin polarization in spin-based sensors to increase their sensitivity to magnetic fields^6–8^, creating spin-based transistors^9^, and replacing slow-switching ferromagnetic data storage with the faster antiferromagnetic storage which uses spin-transfer and spin-orbit torque^3,8,10–14^. The possibility of realizing a nonequilibrium spin polarization led to the emergence of a branch of spintronics – spin-orbitronics, where exotic phenomena like spin-Hall conductivity^15,16^ and spin-galvanic effect^17^ can arise.

Uncovering the complex energy landscape in terms of magnetic states is crucial in accurately modeling the intricate phenomena that propel these technological advances forward, as well as creating innovative materials that can achieve them to the fullest extent.

Density-functional theory (DFT)^18,19^ is one of the staple tools in support of these endeavors, due to its predictive power despite being computationally inexpensive. As the name suggests, DFT is centered around the expression of the total energy of a system as a functional of the charge density; for magnetic systems this functional is typically made to be dependent on the spin density as well^20^. The form of the exact energy functional (which would lead to the exact ground state) remains an open question, and many approximations that perform better or worse for given systems have been created throughout the years. A constant between all these is that, due to the complex interactions between charge and spin densities, the energy landscape harbors multiple local minima and a (potentially degenerate) global minimum^21–30^. Each of these represent one self-consistent solution of the Kohn–Sham (KS) equations^19^, and an associated electronic configuration or state of the system. The ultimate goal of a robust first-principles method should therefore be to identify both the global energy minimum and ground state as well as all other possible solutions, which becomes an increasingly more complex problem to solve in the presence of multiple minima.

Nonetheless, efforts can be undertaken to explore the energy landscape by generating many initial configurations, optimizing the energy of each of them, and assigning the ground state to the local minimum with the lowest total energy. This ability to reliably initialize the state of the system lies at the heart of the current work. In the case of magnetic materials, many of the properties originate from the nature and occupation of the open-shell *d* or *f* orbitals, making them natural proxy variables to characterize the overall state of the system. We have thus devised a method that forces the system into a state that supports a target set of occupied local orbitals. This effectively initializes the electronic state, from which then an unconstrained self-consistent field (SCF) procedure converges towards the closest local minimum.

Previous methods used in similar investigations^28,31–34^ rely on directly replacing the orbital occupations with the target ones, and/or exploit the inclusion of a Hubbard potential to hopefully drive the state of the system towards the target. Not only does this mean that the strength of the driving term depends on the size of the applied Hubbard correction, requiring sweeps of the latter; it also assumes that the form of the Hubbard potential is the correct one to force the system towards the target. To alleviate these issues, the present approach explicitely constructs the correct set of Lagrange multipliers, and their associated energy penalty and potential to drive the system exactly to the target occupation matrices. Moreover, the Lagrange multipliers and the occupations are simultaneously updated during the self-consistent procedure, which makes the approach converge towards the target robustly and efficiently; releasing the constraints then leads to the closest self-consistent minimum.

Using this method as the basis for a global search algorithm, we are able to uncover a complex landscape of self-consistent metastable states for many textbook magnetic systems. Including a Hubbard correction to better describe the localized physics around the magnetic ions leads to a real explosion of the number of independent self-consistent states, as was theoretically postulated^27^. We observe a similar trend when using hybrid functionals (HSE^35^ or PBE0^36^), even though the total number of self-consistent minima is less than with Hubbard-corrected functionals; notably, both hybrid functionals and DFT+U show great similarities in the low-energy spectrum, and are at variance from local/semi-local DFT. The observed complexity of the energy landscape is in agreement with recent extensive quantum chemistry calculations^37^, with the difference that each of the self-consistent states reached by DFT has a single rather than a multi-determinant wavefunction, with no well-defined spin quantum number.

The fact that by only influencing the occupations of localized orbitals we manage to identify a vast number of self-consistent states underlines once more the importance of atomic physics in magnetic systems, and thus the need to accurately describe it. At the same time, our results highlight that one should exercise care when simulating magnetic systems, since a single calculation will only find a single local minimum; whether this corresponds to the ground state depends on implementation details, and the user’s intuition and ability in fine tuning input parameters. The method reported here instead provides a transparent yet powerful avenue to explore in principle all minima without human intervention, and assigns the ground state label to one with the lowest energy. This type of application has become more relevant recently in the context of high-throughput searches, many of which are based on advanced DFT functionals, for promising materials^38–41^, especially those focused on magnetic materials such as in refs. ^42,43^.

We first outline our formulation and implementation, based on Lagrange multipliers and associated constraining energy penalties. This is followed by a demonstration of the effectiveness of the approach in achieving even exotic target occupations and oxidation states. The ability of controlling oxidation states^44–46^ opens the door to investigations of electron-transfer excitations^47^, compounds with mixed valence^48–50^ or charge disproportionation such as skutterudites^51–58^ or, e.g., LiR_2_O_4_ spinels with *R* = (*Mn*, *Fe*) that are of interest as cathodes in batteries^59,60^. We then explore extensively the energy landscape in NiO, a prototype magnetic oxide, by utilizing a global minimization technique. Finally, we discuss the outlook on the potential applications of the methodology and of DFT with Hubbard corrections as a whole. We also present in the Supplementary Information many other case studies, including simple transition-metal oxides, i.e., MnO, FeO, CoO and CrO; skutterudites, i.e., FeSb_3_ and RuSb_3_; monoclinic and rocksalt phases of p-orbital magnetic SrN and bcc-Fe.

## Results

### Methodology

The starting point is the choice of the manifold of local orbitals that are targeted by the constraining potential. Since magnetic materials are the main focus here, we limit the local orbitals to the shell from which magnetism originates. For transition-metal ions this will be the *d*-shell, for actinides and lanthanides (such as uranium and gadolinium, respectively) the *f*-shell is targeted, and for compounds containing molecular magnetism such as SrN, one would target the *p*-shell. In fact, these are exactly the same manifolds often targeted by the Hubbard extensions of DFT, allowing us to repurpose much of the work and tools that were developed in support of DFT + U^61–63^. Of course, other choices of localized orbitals such as maximally localized Wannier functions^64,65^ or other orbitals could be used. After having defined the manifold of local orbitals, the occupation matrices *n* can be constructed from the occupied KS states (see the Supplementary Information).

The objective is to first constrain the system to satisfy a target set of occupation matrices (see Supplementary Eq. (1) for the definition) and to then release the constraints to allow the system to converge freely to the nearest minimum of the unconstrained energy functional. This is achieved by a constrained optimization scheme based on Lagrange multipliers, which we adapt for the self-consistent field (SCF) calculations that form the basis for solving the KS equations in DFT. In essence, we add a penalty to the total energy functional that depends on the distance from the target occupation matrix, and is written in its most general form as:

1 $$\tilde{E}={E}_{DFT}+\mathop{\sum}\limits_{I,\alpha \beta }{\lambda }_{\alpha \beta }^{I}\left({n}_{\alpha \beta }^{I}-{\tilde{n}}_{\alpha \beta }^{I}\right),$$

where ${\lambda }_{\alpha \beta }^{I}$ denote the Lagrange multipliers that represent the constraints.

This leads to an additional potential, applied to the manifold of localized orbitals:

2 $${\tilde{V}}_{\alpha \beta }^{I}=\frac{\partial \tilde{E}}{\partial {n}_{\alpha \beta }^{I}}={\lambda }_{\alpha \beta }^{I}.$$

The *λ* are then optimized together with the other degrees of freedom until self-consistency is reached, i.e. when *n* is within a given threshold to $\tilde{n}$. Afterwards, they are decreased to zero, allowing the system to evolve towards the closest self-consistent energy minimum and associated local occupation matrix. It is clear that in general the final occupation matrix will differ from the target one, which can be thought of as simply an “initial” guide for the system. This underlines again that the final results are self-consistent states of the *unconstrained* energy functional (see the Supplementary Information for ways of specifying the target occupation matrices and implementation details).

Our methodology is very similar to the well-known constrained-DFT (CDFT) method^47,66–69^, where the target is the occupation of certain orbitals rather than amount of charge of spin in a given region of space. In fact, the resemblance is even closer than initially appears in that filling certain orbitals directly corresponds to constraining towards certain distributions of charge or magnetization. The main difference is the specificity of our target, i.e. only the contribution to charge and magnetization of the orbitals in the manifold to which we apply constraints. This also means that using different definitions of the local manifold, e.g. by using different projectors in the DFT+U procedure, does not change the method in itself rather than manipulating and exploring the self-consistent occupations of different orbitals, corresponding to different charge and magnetization distributions. We therefore believe that only changing the local manifold, while keeping all other things equal, will most likely lead to identical final states provided that the local manifold has roughly the same contribution to the energy of the total system.

As a paradigmatic case, we focus in the main text on the well-known antiferromagnetic (AFM) material NiO. We performed searches on the AFM [111] unit-cell of NiO, including two nickel and two oxygen atoms, to gauge the convergence and impact of different functionals and values of U. We then demonstrate the combinatorial explosion of self-consistent states by performing searches on supercells.

### Convergence

To gauge the convergence towards the target occupations, we define a Euclidean distance metric between occupation matrices *n* and ${n}^{{\prime} }$ as:

3 $$d(n,{n}^{{\prime} })=\sqrt{\mathop{\sum}\limits_{I,\alpha \beta }{\left({n}_{\alpha \beta }^{I}-{n}_{\alpha \beta }^{{\prime} I}\right)}^{2}}.$$

**Fig. 1:**
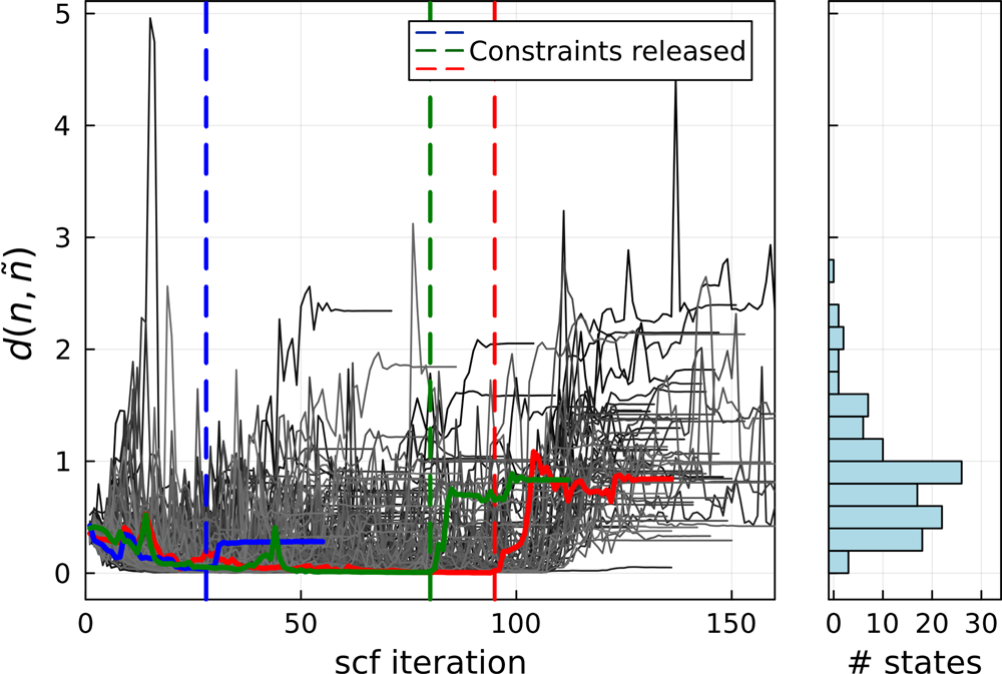
Distance to target occupation matrix during self-consistent cycle. The left panel shows the evolution of $d(n,\tilde{n})$ for ~120 scf calculations, each with a different target $\tilde{n}$ imposed. The red, green and blue graphs highlight three representative examples, with the dashed lines showing where the constraints *λ* were released when *d* < 0.1, after which the system converges to the closest *unconstrained* minimum. The right-hand panel shows $d(n,\tilde{n})$ at the end of the SCF calculation, i.e. after reaching convergence with all *λ*_*αβ*_ = 0. All the calculations shown here converge to a self-consistent state (occasionally after more than 150 SCF steps).

Figure 1 details the evolution of *d* during the various SCF calculations that each constitute a trial with a different target occupation matrix. We can conclude that in general the calculations converge quickly towards the target occupation matrices, highlighting the efficacy of the constraining method. It also highlights that after releasing the constraints the system does not necessarily remain very close to the target occupations, but rather evolves to the nearest self-consistent state that presents a minimum of the energy landscape. Each self-consistent state therefore acts as a funnel pulling nearby constrained solutions towards itself.

### Functionals

While *d* in Eq. (3) is a useful distance metric, it does not take into account the local point group of the atoms whose symmetry operations lead to certain occupation matrices being physically identical even though the distance *d* between them is nonzero. In the following, we therefore use the band distance metric *η*(*A*, *B*) defined in Eq. (5) of ref. ^70^ as a “physically aware” metric for the distance between electronic states *A* and *B*: it represents the average difference of the occupied bands of systems A and B, calculated on the full Brillouin Zone, which is necessarily zero if A and B are symmetry partners.

**Fig. 2:**
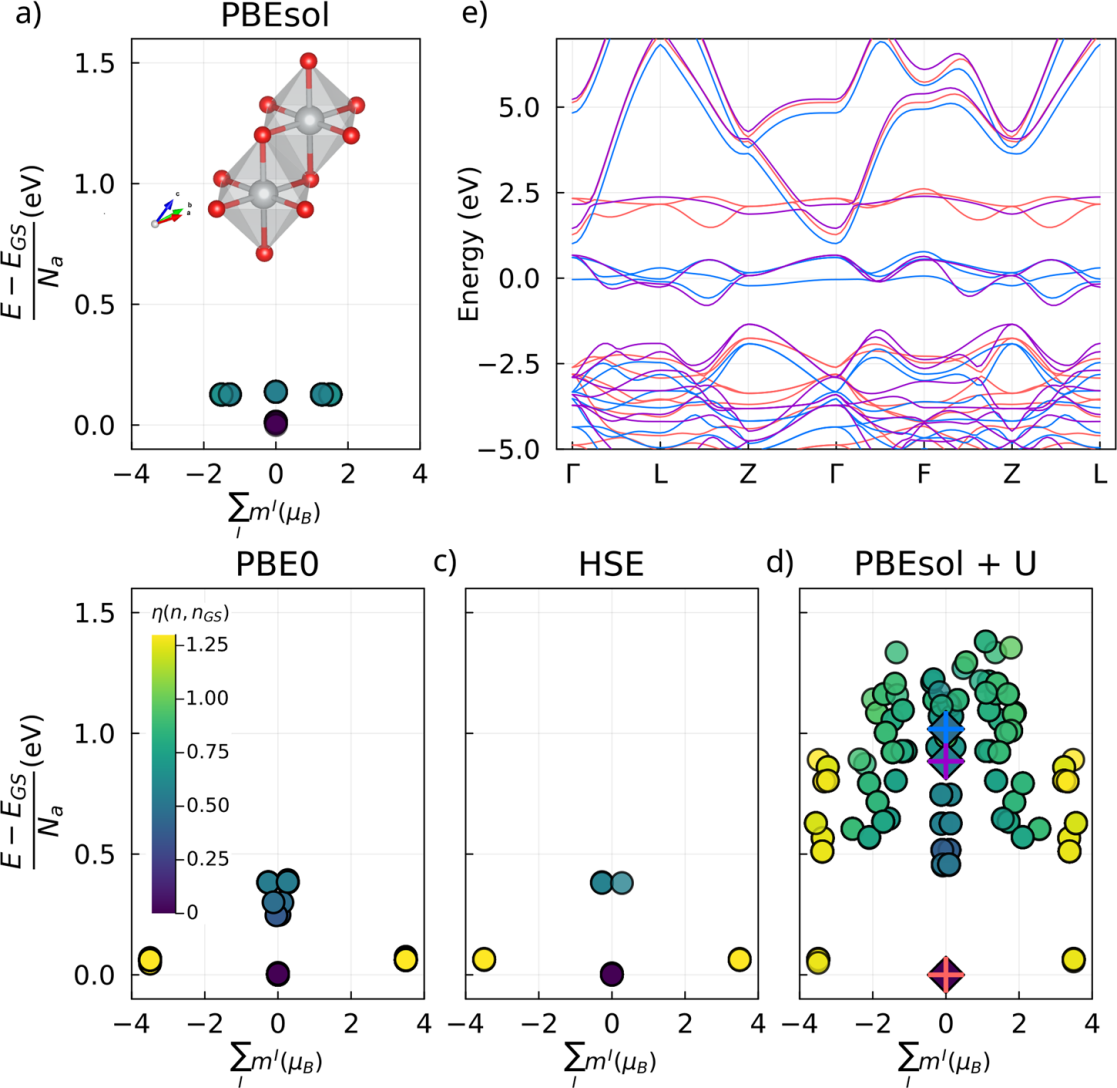
Global search with different functionals for Ni_2_O_2_. Each point in panels **a**–**d** shows a self-consistent state of Ni_2_O_2_, whose structure is shown in the inset of panel **a**, for the four functionals studied. The states correspond to local minima of the original functional *after the constraints are released*, with their color denoting the band distance *η* for the occupied states to the ground state. The x-axis denotes the sum of local atomic moments, each defined as ${m}^{I}=Tr[{n}_{\uparrow }^{I}]-Tr[{n}_{\downarrow }^{I}]$, while the y-axis shows the total energy difference per atom with respect to the ground state, with *N*_*a*_ denoting the number of atoms in the unit cell. Panel **e** shows the band structures of the three states found with the PBEsol + U functional, marked by the corresponding colored crosses in panel **d**. Importantly, PBE0, HSE and PBEsol + U display very similar low-energy solutions.

Four functionals are tested: PBEsol, PBEsol + U, PBE0 and HSE, where we used a U value of 6 eV in the Hubbard-corrected simulations. The impact of U will be discussed in greater detail below. Performing the global search for the four different functionals leads to the results shown in Fig. 2. All functionals tested display a multiplicity of self-consistent states or local minima, with the [111] AFM state being the ground state. We see that the PBEsol simulations in panel a) lead to the least amount of different states, and the absence of solutions with high spin/total magnetization. The hybrid functionals in b) and c) also lead to a relatively low amount of states, with the main difference to PBEsol being that they do find the high-spin states as locally stable solutions. This would be relevant e.g. when using the total energy differences between the ferromagnetic (FM) and AFM configurations to calculate the magnetic exchange parameters of the Heisenberg model^71^.

Including a Hubbard correction in PBEsol leads to a veritable explosion of metastable self-consistent states, as shown in panel d. We can notably observe that the low-energy states found when using hybrid functionals are also present in the Hubbard corrected simulations, highlighting how all these approaches lead to the correct ground state (as long as it can be described by a single determinant of approximate but broadly self-interaction-free DFT). There are, however, many states that display intermediate spins and have higher energies to those found with the other functionals. This is largely due to the shape of the Hubbard correction, as discussed previously^27^, while also leaving open the fascinating question of what would be the case for the exact energy functional/Schrödinger equation. It also highlights that some care needs to be excercised when using Hubbard functionals in the sense that different fillings of the local orbitals might lead to a similar total magnetization but widely different energetics, as demonstrated by the vertical extent of states in Fig. 2 for most given magnetizations.

As shown by the band structures in panel e) of Fig. 2, this can lead to very different band structures even though the magnetic configuration of each of the showcased states is fully AFM. While the ground state has a bandgap as expected, the higher lying states are metallic with the highest energy state (in blue) having three flat bands around the Fermi level and the middle energy state in purple only having two, the last one being shifted further upwards. The valence bands are very similar for the two high energy states, leading to a relatively small *η* between them and thus similar coloring of their respective points in the scatter plot. We note in passing that all panels should be left-right symmetric (for every self-consistent state with ∑_*I*_*m*^*I*^ = *M* there should also be one corresponding to − *M*). This is largely the case, underscoring the reliability of the algorithm in exploring the landscape; see also Supplementary Figs. 1–4.

### Supercells

**Fig. 3:**
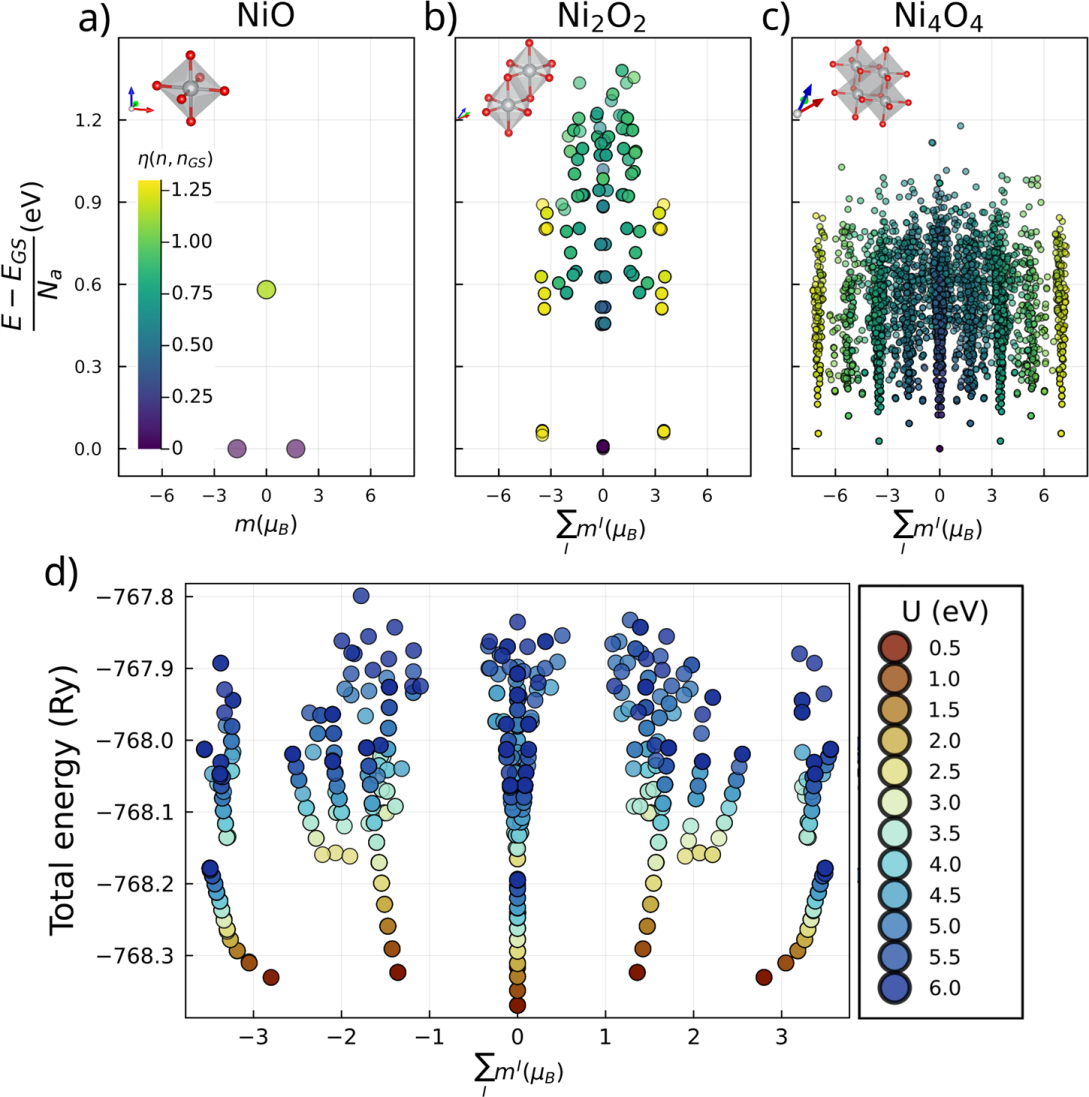
Impact of supercell size and Hubbard U for NiO. The states in panels **a**–**c** are visualized in a similar way to Fig. 2, with the supercell used in the search displayed by the insets. Panel **b** corresponds to panel **d** in Fig. 2. The bottom panel shows how the results change of panel **b** with the value of the Hubbard U parameter.

Next, we investigate the influence of the chosen unit cell on the amount of metastable states found with DFT + U by performing a search on three cells for NiO, shown in the topmost panels of Fig. 3. We will refer to these as NiO, Ni_2_O_2_, and Ni_4_O_4_, signaling the number of atoms inside the supercell. Ni_2_O_2_ corresponds to the results previously discussed in context to Fig. 2, and the smallest one that admits the AFM ground state.

As expected, the number of metastable states explodes combinatorially when increasing the supercell size. Essentially, the ionic physics lead to a number of potential local metastable states which can get stabilized by the states on neighboring ions. Larger supercells allow symmetry breaking, leading to many more metastable states, as opposed to the smaller cells. As shown by the leftmost panel in Fig. 3, a single nickel ion only has two metastable states unless stabilized by neighbors with the appropriate local state. This behavior is only possible in larger unit cells, since otherwise each nickel is necessarily identical. Even in this case, though, there is a combinatorial explosion of metastable states as the supercell size is increased. As demonstrated by the results for MnO (with close to half-filled *d*-shell) in the Supplementary Information, this situation becomes even more remarkable if there are already many metastable states in a single unit cell. Importantly, the oxidation states themselves can also vary accross supercells.

In order to understand the degree to which metastable states are a result of the shape of the Hubbard correction to the energy, we performed simulations for different values of U, using the states found for Ni_2_O_2_ with a U value of 6 eV as targets (top middle panel of Fig. 3). The results are shown in the bottom panel of the same figure. It is clear that lowering the value of U quickly diminishes the amount of metastable states, while also changing their relative energetics. This latter point is quite important since energy differences between some of the metastable states (e.g. FM vs AFM) are often related to the magnon spectrum of the system, highlighting again that it is crucial to use the correct value for U, as advocated in refs. ^72–75^.

## Discussions

We have presented a transparent and effective method to explore the potential energy surface of most (magnetic) materials in supercells of increasing size (for commensurate/incommensurate spin spirals, see ref. ^76^). In combination with a global search algorithm we have uncovered a veritable zoo of metastable electronic states that are all self-consistent solutions to the Kohn–Sham equations that form the basis of DFT. This is a precious capability in the effort to reliably determine ground states of magnetic systems from first-principles without the need for human intuition or experimental input. With the advent of technological applications such as spintronics that are based on complex magnetic behavior it is of paramount importance not only to describe the actual ground state, but also to get access to the properties of the low lying excited states. Our method provides an efficient way to drive ab-initio DFT calculations towards the self-consistent states that represent these excitations, allowing for high-throughput screening of databases with such multi-faceted properties as the target. Furthermore, while it was known that magnetic systems can harbor different local minima, this was never explored as extensively as in the current work. This highlights the potential pitfalls of using single-point calculations as a black box for simulating (magnetic) materials.

## Methods

### Implementation details

We have implemented our method in the open-source Quantum ESPRESSO distribution^77^. In order to explore the complexity of this landscape of metastable self-consistent states, we implemented a global search algorithm using the constraining methodology as a way to initialize the system in a given state. This, and a supporting framework, has been implemented in RomeoDFT. While we defer a more technical analysis of the algorithm, we briefly summarize it here. First, an unconstrained calculation is ran to determine the base number of electrons per ionic site. We then generate ten random trials filling the orbitals with either the same number of electrons, or with one more or one less to capture different oxidation states. Each of these will lead to a local minimum, with some falling into the same local minimum. We take pairs of the occupation matrices corresponding to the unique solutions, and generate “intersection” trials which represent the mean of the occupation matrices in each pair. These are then used as the targets for the next step. This intersection of unique occupation matrices is repeated until the ratio of newly discovered unique states to the number of trials goes below a given threshold, after which the global search concludes.

In all our results, unless stated otherwise, the SSSP-PBEsol v1.1 efficiency library^70^ of pseudopotentials is used. Sampling of the Brillouin Zone is performed on a centered 8 × 8 × 8 Monkhorst–Pack grid and we used wavefunction and density planewave cutoffs of 60.0 Ry and 480.0 Ry, respectively.

### Supplementary information

Supplementary Information

## Data Availability

All data generated in this work is available under request. The datasets used to produce figures are available from Materials Cloud at https://archive.materialscloud.org/record/2024.76.

## Code availability

All software packages used for this work are open-source. The latest stable versions can be downloaded at https://github.com/louisponet/RomeoDFT.jl and https://gitlab.com/louisponet/q-e/-/tree/broyden_constraints.
