# Supplementary material for: The energy landscape of magnetic materials
Source: NPJ Comput Mater. 2024 Jul 16;10(1):151. doi: 10.1038/s41524-024-01310-w (PMC11251989; doi:10.1038/s41524-024-01310-w)
Supplement: Supplementary file 1 — Supplementary Information [file 41524_2024_1310_MOESM1_ESM.pdf]

I. SUPPLEMENTARY INFORMATION

II. FURTHER METHODOLOGICAL BACKGROUND AND IMPLEMENTATION DETAILS

The occupation matrices n that are the core ingredients of our algorithm are constructed by projecting the Kohn-Sham (KS) wavefunctions onto a local basis set [1]:

$$n^I_{\alpha\beta} = \sum_{mk} f_{mk} \langle \psi_{mk} | \phi^I_\alpha \rangle \langle \phi^I_\beta | \psi_{mk} \rangle , \tag{1}$$

where $|\phi^I_\alpha\rangle$ are atomic-like orbitals, and α denotes both the spatial and spin component of the orbitals. In the plane-wave DFT codes that we utilize here these are taken from the atomic orbitals specified in the pseudopotentials, i.e. as a result of solving the Schrödinger equation around the single atoms. The trace is performed over all KS states $|\psi_{mk}\rangle$, which are identified by their band index m and crystalline momentum (k-point in the first Brillouin zone). f_{mk} denotes the occupation of the KS states, which for insulators is always 0 or 1 [2], but for metallic systems can be non-integer. The atomic orbitals of the different sites are in general not orthogonal to each other, thus having nonzero overlap. In order to avoid any double counting in the above definition of the occupation matrix, we first orthogonalize all the atomic orbitals using the Löwdin method [1, 3].

During the SCF cycles, the Lagrange multipliers λ of Eqs. (1-2) in the main text are updated using their gradient:

$$-\frac{\partial \tilde{E}}{\partial \lambda^I_{\alpha\beta}} = n^I_{\alpha\beta} - \tilde{n}^I_{\alpha\beta}, \tag{2}$$

an expression consistent with the intuitive meaning of constraining ("forcing") the system towards a target occupation matrix $\tilde{n}$.

In Eq.(1) target occupations are specified for each element of the occupation matrices, e.g. fifty elements per ion with a valence d-shell. In many cases such granular control is not required or even desired, e.g. for materials where a clear t_{2g}-e_g splitting is expected. In that case, it is not important exactly which local orbitals are occupied, but simply that there is a set of fully occupied and fully empty orbitals leading to a given set of eigenvalues of n.

First, the current occupation matrix is diagonalized:

$$n = VDV^\dagger. \tag{3}$$

Then the eigenvalues in D are replaced with the target ones, and the full target occupation matrix is reconstructed:

$$\tilde{n} = V\tilde{D}V^\dagger. \tag{4}$$

More explicitely:

$$D = \begin{pmatrix} \eta_1 & 0 & \cdots & 0 \\ 0 & \eta_2 & \cdots & 0 \\ \vdots & \vdots & \ddots & \vdots \\ 0 & 0 & \cdots & \eta_n \end{pmatrix}, D_t = \begin{pmatrix} \tilde{\eta}_1 & 0 & \cdots & 0 \\ 0 & \tilde{\eta}_2 & \cdots & 0 \\ \vdots & \vdots & \ddots & \vdots \\ 0 & 0 & \cdots & \tilde{\eta}_n \end{pmatrix}, \tag{5}$$

This $\tilde{n}$ can then be inserted in Eq.(1) to define the constrained functional. In practice, the eigenvectors (or orbitals) of the occupation matrix obtained at the first self-consistent iteration are used for V. While this seems like a relatively arbitrary choice, these orbitals indirectly result from a diagonalization of the initial Hamiltonian and therefore already include information such as the symmetry of the system and the crystal field of surrounding atoms, and should thus not be far removed from physically reasonable orbitals.

As mentioned above Eq.(1), we have chosen to utilize the same local orbital manifold as in the DFT + U scheme [4], meaning that the contribution to the potential originating from the Lagrange multipliers (Eq.(2)) can be simply added to the Hubbard correction, leading to the total local potential:

$$V^I_{\alpha\beta} = U\left[\frac{\delta_{\alpha\beta}}{2} - n^I_{\alpha\beta}\right] + \lambda^I_{\alpha\beta}. \tag{6}$$

This means that the method can be implemented without altering anything fundamental in the underlying DFT code. It is, however, crucial that the Lagrange multipliers λ are updated using the same method as for the density and other quantities that determine the self-consistent potential, since it implicelty determines the occupation matrices. If this is not done, the calculations converge either extremely slowly or not at all.

III. GLOBAL SEARCH RESULTS FOR SELECTED MATERIALS

A. Simple Transition-Metal Oxides

The observations discussed in the main text are obviously not limited to NiO; we present here global searches for four other single-ion transition-metal oxides: MnO, FeO, CoO and CrO. As shown in Supplementary Figure 1, we find that all display the same phenomenology, with the differences highlighting again that the atomic physics as the main driver of the phenomenology.

MnO – MnO is a standard superexchange-mediated type-II AFM insulator [5–11], with alternating FM planes stacked along the cubic [111] direction [12]. Bulk AFM MnO has a rhombohedral $R\overline{3}m$ structure which changes to paramagnetic rocksalt $Fm\overline{3}m$ above its Néel temperature of 118 K [6, 13]. Looking at the results in Supplementary Figure 1, the main difference with the NiO discussed in the main text is that already on the single ion level there are many more metastable states with

varying filling of the local orbitals. This leads to an even more severe combinatorial explosion in supercells.

FeO – As MnO, FeO also shows a transition from AFM order at low temperature [13] to paramagnetism with rocksalt structure [7] above the Néel temperature of 198 K [14]. Being stable at high pressure, FeO may have an important implication in the earth science because, given that it is one of the basic oxide components in the interior of the Earth [15, 16]. In FeO, the orbital-ordering induced Jahn-Teller effect makes a stronger tetragonal or orthorhombic distortion superimposing to the rhombohedral one [12]. While we study here only the high-symmetry crystal structure of FeO, we expect that upon relaxation of the different metastable states in a supercell one could recover the Jahn-Teller distortion.

The panels of the second row of Supplementary Figure 1 show that for FeO largely the same statements can be made as in the case for MnO. The main difference is that there are now quite some states that are very close to, but distinguishable from, the ground state. This leads to a cluster of five states with total energy per atom within 0.1 eV of the lowest energy state for the single Fe ion unit cell. Moreover, we note that using the standard implementation of DFT + U in `Quantum ESPRESSO`, we do not converge to the ground state but rather to the highest energy state in the cluster as shown by the red cross. This highlights how one should exercise care in understanding when the ground state is reached.

CoO – High-resolution synchrotron powder diffraction experiments in the temperature range 10–300 K were used to identify the monoclinic symmetry (C2/m space group) of AFM CoO [17]. The magnetic ordering in this material is associated with a cubic-to-monoclinic transition that is, thus, of first order [17]. No first-order discontinuity and no second phase transition close to Néel temperature was observed both with highly sensitive birefringence and VSM/MPMS magnetometers measurements [18, 19]. Magnetic measurements reveal the presence of FM interactions at low temperatures in small CoO nanoparticles (¡16 nm) [20]. The electronic-structure of CoO has been investigated using x-ray photoemission spectroscopy (XPS), bremsstrahlung isochromat spectroscopy (BIS), and x-ray-absorption spectroscopy [21], showing that CoO is a highly correlated insulator with electronic band gap of 2.5±0.3 eV [21]. The first ionization state of CoO is therefore of strongly mixed Co $3d$ and 0 $2p$ character. Similar to our results, DFT+U calculations have confirmed the Mott insulating behavior with associated AFM spin ordering [22].

CrO – A comprehensive theoretical and experimental investigation of CrO_n^- (n = 1–5) and their corresponding neutral species was carried out by Gutsev *et al.*. The photodetachment photoelectron spectra and the calculated adiabatic and vertical binding energies show that only one stable structure was predicted for CrO_5^-.

NiO – NiO is a well-known Mott-charge-transfer system with an fcc crystal structure and AFM magnetic configuration below the Néel temperature of 523 K [7, 23, 24].

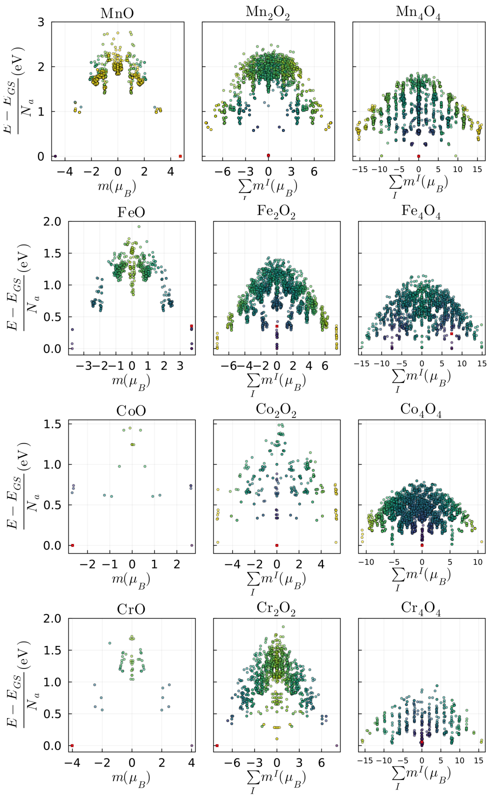

Supplementary Figure 1: **Metastable states for supercells of different oxides.** The states are displayed in the same form as panels (b-d) of Fig.2 in the main text. The red crosses in each plot signify the result that is achieved by running standard QE with the magnetic configuration that was found as the ground state by the global search algorithm.

Interestingly, superparamagnetic or FM behaviors were observed by experimental measurements of the magnetization in NiO nanoparticles [25–28], with AFM aligned core spins and a spin glass like surface layer.

B. Skutterudites

Skutterudites are highly promising functional materials for thermoelectric and spintronic applications. They exhibit a complex phenomenology both in spin configurations and charge disproportionation [29]. Skutterudites crystallize in a body-centered cubic (bcc) structure belonging to the $Im\bar{3}$ space group. Such a structure is known for the presence of an empty space that can be filled with rare earths [30, 31], lanthanides [30] or alkali metals [32]. This filler atom gives raise to the so-called filled skutterudites, whose thermal properties have been subject of numerous experiments [30, 31, 33–37].

While $FeSb_3$ has been synthesized and its magnetic properties have been studied in detail, $RuSb_3$ has been synthesized but a magnetic characterization of the ma-

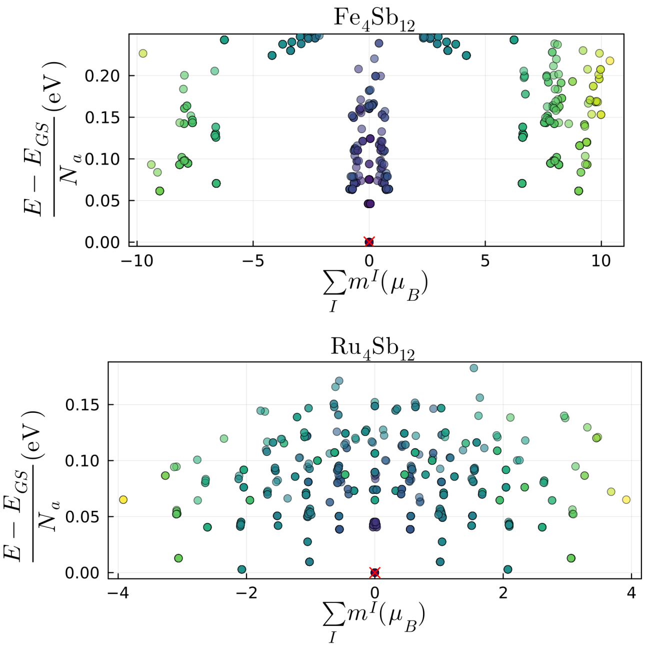

Supplementary Figure 2: **Metastable states of $FeSb_3$ and $RuSb_3$.** The states are displayed in the same form as panels (b-d) of Fig.2 in the main text. The red crosses in each plot signify the result that is achieved by running standard QE with the magnetic configuration that was found as the ground state by the global search algorithm.

terial has not yet been carried out. $FeSb_3$ is a paramagnetic tiny gap semiconductor at room temperature [38], while its low temperature phase is found by first-principles calculations to be AFM [39]. $RuSb_3$ exhibits similar features but experiments highlight the fact that its decomposition is exothermic causing it to be thermodynamically metastable at all temperatures [40]. The results of performing the global search on these materials is shown in Supplementary Figure 2. The importance of allowing for different oxidation states is highlighted by the fact that Fe and Ru have mixed-valence in these materials.

C. SrN

Magnetism in systems that do not contain transition-metal or rare earth ions was recently observed or predicted to exist in a wide variety of systems [41–44]. In ideal bulk II–V and II–IV compounds, such as molecular crystals constituted by O_2 or N_2 molecules, the non-vanishing spin polarization originates from the $2p$ shell of light atoms (anions). This happens predominantly for ions from the second row of the periodic table, which have a Hund‘s rule energy close to that of transition-metal atoms [45]. This explains why only crystals that contain C, N and O are expected, and found, to be spin-

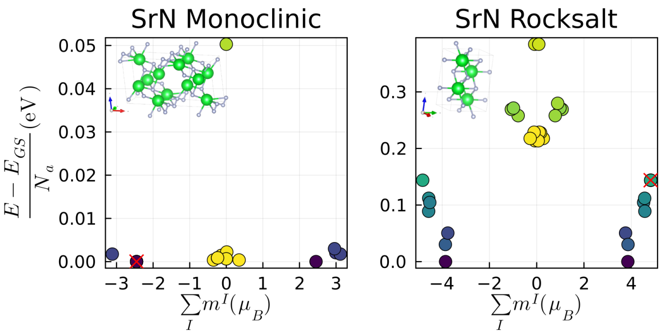

Supplementary Figure 3: **Metastable states of SrN.** The two unit cells are shown as insets in each panel. The states are displayed in the same form as panels (b-d) of Fig.2 in the main text. The red crosses in each plot signify the result that is achieved by running standard QE with the magnetic configuration that was found as the ground state by the global search algorithm.

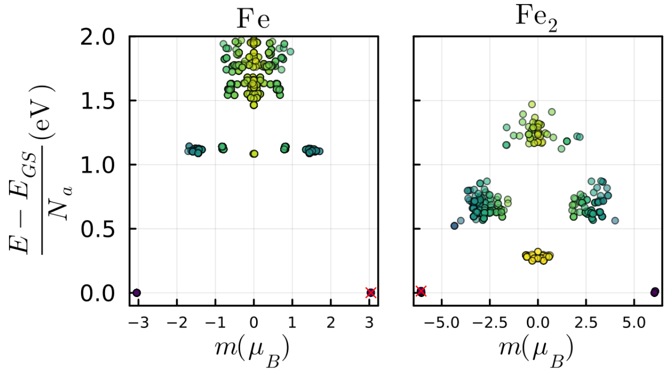

Supplementary Figure 4: **Metastable states for bcc iron.** The states are displayed in the same form as panels (b-d) of Fig.2 in the main text.

polarized. As an example of this class of materials, we study the metastble states of SrN in two structural configurations: monoclinic and rocksalt, both observed by experiments [46–48]. Previous electronic first-principles tudies have found that the rocksalt phase is a stable FM half-metal [49, 50].

D. bcc Fe

To further verify that the phenomenology also appears for metallic systems, we ran a global search on bcc-Fe, both in single and doubled unit cell configurations. As Supplementary Figure 4 demonstrates, the results are in line with the other systems previously discussed.

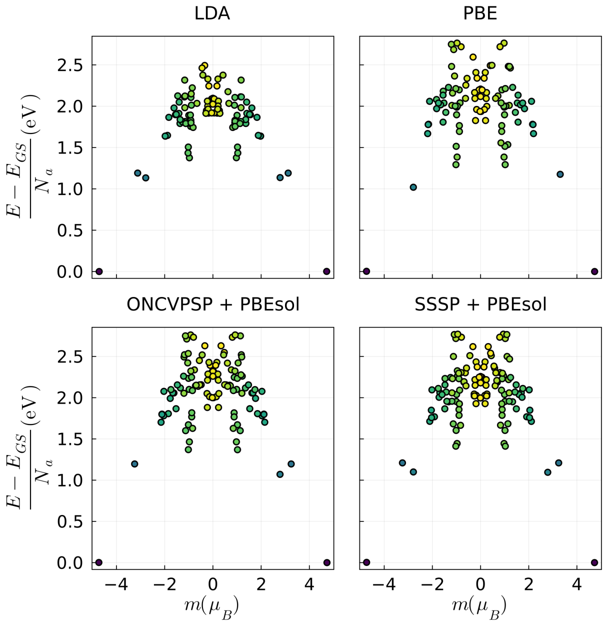

Supplementary Figure 5: **Influence of pseudopotentials or functionals in MnO.** Global searches based on a unit cell with a single Mn and O atom with different combinations of pseudopotentials were performed. The states are displayed in the same form as panels (b-d) of Fig.2 in the main text. The bottom right panel corresponds to the top leftmost panel of Supplementary Figure 1

IV. INFLUENCE OF PSEUDOPOTENTIALS

We have verified that the results do not change markedly when using different Hubbard corrected functionals in combination with different functionals, by running a global search for MnO using four different combinations: SSSP + PBEsol [51], ONCVPSP + PBEsol [52], GBRV + PBE and GBRV + LDA [53]. As shown in Supplementary Figure 5 in all cases we find similar results.

V. HUND'S RULE

We can further rationalize the broad behavior of the energy versus magnetization plots in terms of the ionic magnetization. Indeed, as demonstrated by the fits to a parabola shown by the black graphs in Supplementary Figure 6, the energy versus magnetization distribution follows the shape of Hund's rule energy $E_H = -\gamma S^2$, with S^2 denoting the on-site spin and γ the strength. The values of γ for each of the oxides is shown in the table. The Hund's rule energy penalty is larger in the case of antiparallel on-site spins ($|m| = 0$) since they are allowed to occupy the same spatial orbital thus increasing the Coulomb repulsion energy. It then stands to reason that the inclusion of a Hubbard correcting energy term which leads to more localized spatial orbitals causes a higher energy penalty, which is demonstrated by the trend shown in the bottom panel of Fig.2 in the main text.

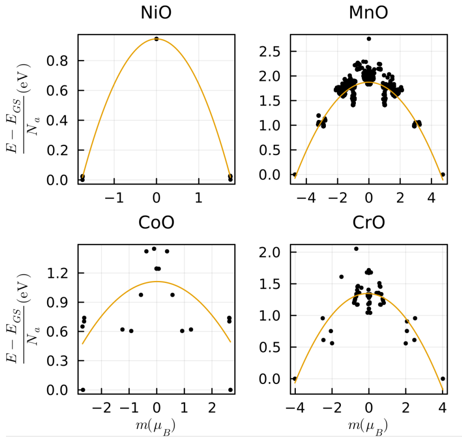

	γ (meV/μ_b^2)
MnO	0.088
CrO	0.091
NiO	0.303
CoO	0.088
FeO	0.095

Supplementary Figure 6: **Hund's rule energy.** The black dots in each of the plots denote the metastable states, with the orange parabola a fit to $-\gamma m^2$. The values of γ for each of the oxides is reported in the table on the bottom right.

[1] C. Tablero, Representations of the occupation number matrix on the lda/gga+u method, Journal of Physics Condensed Matter **20**, 10.1088/0953-8984/20/32/325205 (2008).
[2] When certain smearing techniques are used to improve convergence, the occupations can vary slightly from 0 or 1.
[3] I. Timrov, F. Aquilante, L. Binci, M. Cococcioni, and N. Marzari, Pulay forces in density-functional theory with extended hubbard functionals: From nonorthogonalized to orthogonalized manifolds, Physical Review B **102**, 10.1103/PhysRevB.102.235159 (2020).
[4] I. Timrov, N. Marzari, and M. Cococcioni, Self-consistent hubbard parameters from density-functional perturbation theory in the ultrasoft and projector-augmented wave formulations, Physical Review B **103**, 45141 (2021).
[5] A. Fujimori and *et al.*, Electronic structure of mno, Physical Review B **42**, 7580 (1990).
[6] C. G. Shull, W. Strauser, and E. Wollan, Neutron diffraction by paramagnetic and antiferromagnetic substances, Physical Review **83**, 333 (1951).

[7] W. Roth, Magnetic structures of mno, feo, coo, and nio, Physical Review **110**, 1333 (1958).

[8] M. Lines and E. Jones, Antiferromagnetism in the face-centered cubic lattice. ii. magnetic properties of mno, Physical Review **139**, A1313 (1965).

[9] T. McGuire and R. Happel, The magnetic susceptibility of an mno single crystal, J. phys. radium **20**, 424 (1959).

[10] P. W. Anderson, Antiferromagnetism. theory of superexchange interaction, Physical Review **79**, 350 (1950).

[11] P. W. Anderson, Theory of magnetic exchange interactions: exchange in insulators and semiconductors, in *Solid state physics*, Vol. 14 (Elsevier, 1963) pp. 99–214.

[12] A. Schrön, C. Rödl, and F. Bechstedt, Crystalline and magnetic anisotropy of the 3 d-transition metal monoxides mno, feo, coo, and nio, Physical Review B **86**, 115134 (2012).

[13] H. Shaked, J. Faber Jr, and R. Hitterman, Low-temperature magnetic structure of mno: a high-resolution neutron-diffraction study, Physical Review B **38**, 11901 (1988).

[14] C. McCammon, Magnetic properties of fexo (x¿ 0.95): Variation of néel temperature, Journal of Magnetism and Magnetic Materials **104**, 1937 (1992).

[15] K. Ohta and *et al.*, Highly conductive iron-rich (mg, fe) o magnesiowüstite and its stability in the earth's lower mantle, Journal of Geophysical Research: Solid Earth **119**, 4656 (2014).

[16] R. A. Fischer and *et al.*, Equation of state and phase diagram of feo, Earth and Planetary Science Letters **304**, 496 (2011).

[17] W. Jauch, M. Reehuis, H. Bleif, F. Kubanek, and P. Pattison, Crystallographic symmetry and magnetic structure of coo, Physical Review B **64**, 052102 (2001).

[18] K. Germann, K. Maier, and E. Strauss, Linear magnetic birefringence in transition metal oxides: Coo, physica status solidi (b) **61**, 449 (1974).

[19] L. Zhang, D. Xue, and C. Gao, Anomalous magnetic properties of antiferromagnetic coo nanoparticles, Journal of magnetism and magnetic materials **267**, 111 (2003).

[20] M. Ghosh, E. Sampathkumaran, and C. Rao, Synthesis and magnetic properties of coo nanoparticles, Chemistry of Materials **17**, 2348 (2005).

[21] J. a. Van Elp, Electronic structure of coo, li-doped coo, and licoo 2, Physical Review B **44**, 6090 (1991).

[22] M. J. Han and J. Yu, Electronic structure and magnetic properties of wurtzite coo, Journal of the Korean Physical Society **48**, 1496 (2006).

[23] M. T. Hutchings and E. Samuelsen, Measurement of spin-wave dispersion in nio by inelastic neutron scattering and its relation to magnetic properties, Physical Review B **6**, 3447 (1972).

[24] W. L. Roth and G. A. Slack, Antiferromagnetic structure and domains in single crystal nio, Journal of Applied Physics **31**, S352 (1960).

[25] S. A. Makhlouf, F. Parker, F. Spada, and A. Berkowitz, Magnetic anomalies in nio nanoparticles, Journal of applied physics **81**, 5561 (1997).

[26] Y. Ichiyanagi and *et al.*, Magnetic properties of nio nanoparticles, Physica B: Condensed Matter **329**, 862 (2003).

[27] A. Ngo, P. Bonville, and M. Pileni, Nanoparticles of: Synthesis and superparamagnetic properties, The European Physical Journal B-Condensed Matter and Complex Systems **9**, 583 (1999).

[28] S. Tiwari and K. Rajeev, Magnetic properties of nio nanoparticles, Thin Solid Films **505**, 113 (2006).

[29] E. Di Lucente, M. Simoncelli, and N. Marzari, Crossover from boltzmann to wigner thermal transport in thermoelectric skutterudites, Physical Review Research **5**, 033125 (2023).

[30] B. C. Sales, Filled skutterudites, Handbook on the physics and chemistry of rare earths **33**, 1 (2003).

[31] G. Nolas, G. Slack, D. Morelli, T. Tritt, and A. Ehrlich, The effect of rare-earth filling on the lattice thermal conductivity of skutterudites, Journal of Applied Physics **79**, 4002 (1996).

[32] A. Leithe-Jasper and *et al.*, Ferromagnetic ordering in alkali-metal iron antimonides: N a f e 4 s b 12 and k f e 4 s b 12, Physical review letters **91**, 037208 (2003).

[33] G. S. Nolas, J. Cohn, and G. Slack, Effect of partial void filling on the lattice thermal conductivity of skutterudites, Physical Review B **58**, 164 (1998).

[34] B. Sales, B. Chakoumakos, and D. Mandrus, Thermoelectric properties of thallium-filled skutterudites, Physical Review B **61**, 2475 (2000).

[35] P. Qiu and *et al.*, High-temperature electrical and thermal transport properties of fully filled skutterudites rfe4sb12 (r= ca, sr, ba, la, ce, pr, nd, eu, and yb), Journal of Applied Physics **109**, 063713 (2011).

[36] T. He, J. Chen, H. D. Rosenfeld, and M. Subramanian, Thermoelectric properties of indium-filled skutterudites, Chemistry of materials **18**, 759 (2006).

[37] X. Shi and *et al.*, Multiple-filled skutterudites: high thermoelectric figure of merit through separately optimizing electrical and thermal transports, Journal of the American Chemical Society **133**, 7837 (2011).

[38] A. Möchel and *et al.*, Lattice dynamics in the fesb 3 skutterudite, Physical Review B **84**, 064302 (2011).

[39] E. Di Lucente, M. Simoncelli, and N. Marzari, Crossover from boltzmann to wigner thermal transport in thermoelectric skutterudites, Phys. Rev. Res. **5**, 033125 (2023).

[40] A. L. Smalley, M. L. Jespersen, and D. C. Johnson, Synthesis and structural evolution of rusb3, a new metastable skutterudite compound, Inorganic chemistry **43**, 2486 (2004).

[41] M. Geshi, K. Kusakabe, H. Nagara, and N. Suzuki, Synthetic ferromagnetic nitrides: First-principles calculations of can and srn, Phys. Rev. B **76**, 054433 (2007).

[42] O. Volnianska and P. Bogusławski, Magnetic and structural properties of $II^A{-}v$ nitrides, Phys. Rev. B **75**, 224418 (2007).

[43] H. Peng and *et al.*, Origin and enhancement of hole-induced ferromagnetism in first-row d^0 semiconductors, Phys. Rev. Lett. **102**, 017201 (2009).

[44] H. Peng and *et al.*, Origin and enhancement of hole-induced ferromagnetism in first-row d^0 semiconductors, Phys. Rev. Lett. **102**, 017201 (2009).

[45] O. Volnianska and P. Boguslawski, Magnetism of solids resulting from spin polarization of p orbitals, Journal of Physics: Condensed Matter **22**, 073202 (2010).

[46] J. Gaude, P. L'Haridon, Y. Laurent, and J. Lang, Le système strontium-azote. ii. sur une nouvelle combinaison du strontium et de l'azote, Rev. Chim. Miner **8**, 287 (1971).

[47] G. Auffermann, Y. Prots, and R. Kniep, Srn and srn2: Diazenides by synthesis under high n2-pressure, Angewandte Chemie International Edition **40**, 547 (2001).

[48] G. Auffermann, U. Schmidt, B. Bayer, Y. Prots, and R. Kniep, Speciation of nitrogen–[n3–] and [n2 2–]–in binary compounds, Analytical and bioanalytical chemistry **373**, 880 (2002).

[49] O. Volnianska and P. Bogusławski, Magnetic and structural properties of ii a- v nitrides, Physical Review B **75**, 224418 (2007).

[50] M. Geshi, K. Kusakabe, H. Nagara, and N. Suzuki, Synthetic ferromagnetic nitrides: first-principles calculations of can and srn, Physical Review B **76**, 054433 (2007).

[51] G. Prandini, A. Marrazzo, I. E. Castelli, N. Mounet, and N. Marzari, Precision and efficiency in solid-state pseudopotential calculations, npj Computational Materials **4**, 72 (2018).

[52] D. Hamann, Optimized norm-conserving vanderbilt pseudopotentials, Physical Review B **88**, 085117 (2013).

[53] K. F. Garrity, J. W. Bennett, K. M. Rabe, and D. Vanderbilt, Pseudopotentials for high-throughput dft calculations, Computational Materials Science **81**, 446 (2014).
